# Is This True?

**Published:** 1887-03

**Authors:** C. R. E. Koch

**Affiliations:** Chicago, Ill.


### IS THIS TRUE?

BY DR. C. R. E. KOCH, CHICAGO, ILL.

In looking over some recent numbers of the *Monatsschrift fur Zahnheilkunde,* I was astonished at the statements contained in an article by Zahnarzt Dr. F. A. Heinrich, Frankfort-on-the-Main, entitled, "How One Obtains the Title of Doctor of Dental Surgery in America." The following is a hurried translation of it. It is a serious accusation brought against the moral honesty of our American dental educators. If not sustained by the facts, it is a villainous libel upon American dental colleges in general, and one of them in particular.

We, here in this country, are laboring under the belief that since the formation of the Association of College Faculties, all the abuses formerly practiced and complained of have been remedied, and are now impossible. If there be nothing on which to found such an article, this charge against the good name of American dental science and its educators should not be allowed to circulate in Germany, and to create a false impression as to the real state of affairs of dental education in the United States.

Perhaps it may be well for us, occasionally, to see ourselves as others see us, but certainly the reflections cast in the picture drawn by Dr. Heinrich are not at all flattering. He says:

"Attention has frequently been called, in the literature of our profession, to the exceeding negligence of American dental colleges in enforcing their requirements for obtaining the degree of Doctor.

This is especially so as relates to Germans. As is well known, students are admitted for examination after only a few weeks' attendance (about eighteen or twenty weeks, from which Christmas and New Year's vacation must be deducted), and a diploma is issued. Many of these doctors can scarcely understand a word of English; the examination is, notwithstanding, conducted in the English language. Unfortunately these examinations are not held publicly, but every candidate is examined separately. Whether a third person is present as interpreter is not divulged by the candidates.

"I have formerly called attention to the perfect ease with which a house servant or porter of a dentist, with a certificate of five years of such employment, can pass himself in America as a 'five years' man;' that is, one who has been in the practice of the art and science of dentistry for five years. It is a well-known fact in professional circles, that in Germany, even now, persons improperly practice dentistry, being embellished with the title of Doctor; improperly, because by reason of the liberty of pursuit (*gerwerbe freiheit*) the practice of the healing art (*aertzliche praxis*) is permitted to every one.

"The truth of the above statements has frequently been questioned, but I need only to mention that such certificates were not examined as to their contents in America, but it seemed sufficient that the authorities had authenticated them. What is contained in them is non-essential. If the seal of a police magistrate is upon it—which, in most instances, only authenticates the genuineness of the signature of the maker of the certificate—the gentlemen of the colleges accept as correct and truthful the statements which are made to them by many Germans relating to the nature of the certificate. The signature of the police authorities is upon it, and by the side of it the great seal ! !

"The attacks upon American schools made by Germans, and afterward taken up by the American professional journals, have encouraged several colleges to require a knowledge of the English language as a condition for examination—the Philadelphia College for one. What is the result of this requirement? During this year not a single German has received the doctorate there. Is this merely accidental, or does the college mean to preserve its well-known and valued reputation? In addition, the candidate is required to remain two years at the college. But how is this matter disposed of in the Pennsylvania College? The following case shall teach us. It is so characteristic and striking that it deserves to be made public in order that for once the German authorities may take a decided stand against the continuously growing number of tooth workers (or dentists, as they call themselves), who are only graduated in America.

Herr N. of W. went to America in the fall of 1884 to obtain the dignity of Doctor of Dental Surgery, and returned in the summer of 1885 to Germany, and *without the possession of a diploma.* He was said to have a certificate setting forth that he had been examined, and that (amazing!) at the end of February, 1886, a decision would be rendered as to whether he had passed or not. Several months ago (in the winter of 1885-86), Herr N. in W. was convicted of the unauthorized use of the title of Doctor by a sheriff's court, in three cases, and fined ten, fifteen and twenty marks. According to this, Herr N. was in Germany in the winter of 1885-1886. Singularly, however, the name of Herr N. is on the list of the graduates of February 28, 1886, notwithstanding his presence in Germany. We ask how is this possible? Are there two persons of the same name? No. Perhaps the Doctor title was bestowed *in absentia?* No; for it is specifically stated in the requirements for graduation that this college grants no diplomas *in absentia.* The matter can be explained very simply: Herr N. sojourned one winter in America and then was returned to Germany without a diploma. His name is continued as a participant of next winter's course, and he is found worthy at the end of the term of 1885-86 to receive the diploma. He could not take the diploma with him, as he could not receive it at an earlier date, because the condition of two years' attendance had not been satisfied.

Foreigners in America, then, may learn the highly praised American dental science and dental art by attendance during eighteen weeks, and having their names continued on the lists a year longer. It is the duty of every assiduous dentist to fight continuously against this sort of swindle, and to labor in the direction of putting an end to this mischief of American education.

"Every approbated physician (*Arzt*) has the right to call himself eye, ear, or female doctor. But if he would be an approbated tooth doctor (*zahnarzte*), he must first finish his studies in the first, third and fourth sections of examinations. A practical knowledge of the manufacture of a single artificial tooth and the construction of an entire set is required of him, as well as of the entire technical portion of dental science and the use of the different dental instruments, etc. This is demanded of an approbated physician, and yet others entirely uneducated, but ornamented with a foreign title, are authorized to practice the healing art."

